# RNA binding protein Nova1 promotes tumor growth in vivo and its potential mechanism as an oncogene may due to its interaction with GABA_A_ Receptor-γ2

**DOI:** 10.1186/s12929-016-0288-6

**Published:** 2016-10-12

**Authors:** Yi-An Zhang, Hai-Ning Liu, Ji-Min Zhu, Dan-Ying Zhang, Xi-Zhong Shen, Tao-Tao Liu

**Affiliations:** 1 Department of Hematology, Zhongshan Hospital of Fudan University, Zhongshan Hospital, No.180 Fenglin Road Xuhui District, Shanghai, China; 2 Department of Gastroenterology, Zhongshan Hospital of Fudan University, Zhongshan Hospital, No.180 Fenglin Road Xuhui District, Shanghai, China; 3 Key Laboratory of Medical Molecule Virology, Ministry of Education and Health, Shanghai Institute of Liver Diseases, Zhongshan Hospital, No.180 Fenglin Road Xuhui District, Shanghai, China

**Keywords:** Nova1, GABA_A_ Receptor-γ2, Oncogene, RNA binding protein

## Abstract

**Background:**

The mechanism of Nova1’s role in hepatocellular carcinoma has not been delineated. Also its interaction with GABA_A_ receptor γ2 in HCC is unveiled. This study is aimed to make it clear the distribution, prognostic value of GABA_A_Rγ2 in human hepatocellular carcinoma. And its role in HCC tumorigenesis under the regulation of its alternative splicing factor Nova1.

**Methods:**

Immunohistochemistry staining was used to investigate the distribution and clinical significance of GABA_A_Rγ2 protein expression in hepatocellular carcinoma. In vivo tumorigenticity test was conducted in nude mice by regulation the expression of Nova1. Later, western blot and co-immunoprecipitation were carried out to verify the interaction between Nova1 and GABA_A_Rγ2 in HCC tissue.

**Results:**

Immunohistochemical staining showed GABA_A_Rγ2 expression in HCC. Survival analysis showed intratumoral GABA_A_Rγ2 was an independent prognostic factor for overall survival (OS) and disease free survival (DFS). Up-regulation of Nova1 expression promotes subcutaneous HCC growth in nude mice and western blot showed the ectopic expression of Nova-1 restro-regulates the expression of GABA_A_Rγ2 and GABA. Protein level interaction of GABA_A_Rγ2 and Nova-1 was evidenced by co-immunoprecipitation.

**Conclusions:**

Nova1 interacts with GABA_A_Rγ2 not only in CNS but also in HCC. Nova1’s potential mechanism as an oncogene may due to its interaction with GABA_A_ Rγ2. A better understanding of the mechanism of Nova1 for HCC progression provides a novel target for an optimal immunotherapy against this fatal malignancy.

## Background

Hepatocellular carcinoma (HCC) is the second leading cause of cancer-related deaths worldwide. Although large efforts have been made to have a deeper insight into its pathogenesis and treatment, it is still one of the few cancers with a continued increase in incidence observed over these decades [1].

In recent years, the roles of RNA-binding proteins (RBPs) in the progression and prognosis of HCC have been delineate [2–4]. Nova-1, a neuron specific RNA binding protein, influences ligand-binding, signaltransducing and electrophysiological properties [5]. Our previous study demonstrated the expression of Nova-1 in HCC and proved that high expression of Nova-1 is associated with poor prognosis of HCC [6]. Previous researchers also found that Nova-1 regulates the alternative splicing of GABA_A_ receptor γ2 (GABA_A_Rγ2) pre-mRNA in central nervous system [7]. GABA_A_Rγ2 belongs to the GABA receptor family. GABA_A_ is most prevalent not only in central nervous system but also in peripheral tissues [8]. Activation of GABA_A_ receptors resulted in increased chloride ion influx and hyperpolarization of cell membranes by diminishing the chance of a successful action potential occurring [8]. Recently, lots of reports have pointed out that the relationship between decreased hepatocyte membrane potential and proliferation of hepatocellular carcinoma. Related receptors include GABA_A_ receptor-β3 [9], GABA_A_ receptor-α3 [10], and GABA_B_ Receptor family [11]. However, the potential effects of GABA_A_Rγ2 on tumor proliferation has not been mentioned yet.

In this study we first conducted a tumorigenticity test in nude mice and found that Nova1 could improve tumor growth in vivo. The interaction between Nova1 and GABA_A_Rγ2 was further confirmed by co-immunoprecipitation. Later, verification of the GABA_A_Rγ2 expression in hepatocellular carcinoma and its relations with HCC prognosis were also investigated. After all we demonstrated that Nova1 could promote cell proliferation in vivo, its potential mechanism as an oncogene may related to its interaction with GABA_A_Rγ2, and suggested that Nova1 and GABA_A_Rγ2 could be a novel predictor for HCC recurrence after curative resection.

## Methods

### Cells, animals and reagents

Two human HCC cell lines were used in this study. SMMC7721, Huh7 were purchased from Shanghai Institute of Cell Biology, Chinese Academy of Sciences (Shanghai, China). SMMC7721 was cultured in RPMI medium 1640 (GIBCO, USA). Huh7 were cultured in DMEM supplemented with 10 % fetal bovine serum (GIBCO, Austria). Cells were cultured and passaged in 37 °C incubator with humidified atmosphere containing 5 % CO_2_. Protocols of the establishment of cell lines to induce the Nova1 expression or silencing was summarized in previous study [6]. BALB/c nude mice were purchased from Shanghai Experimental Animal Centre, Chinese Academy of Science. Experimental animals were kept in the central animal facility of the Zhongshan Hospital affiliated to Fudan University and housed in laminar-flow cabinets under specific pathogen-free conditions. All studies on mice were conducted in accordance with the National Institute Guide for the Care and Use of Laboratory Animal. Goat anti-human Nova1 polyclonal antibody was purchased from LifeSpan Biosciences (Seattle, USA). Rabbit anti-human GABA_A_Rγ2 antibody was from Millipore. Rabbit anti-human GABA antibody was from Abcam. HRP affinipure goat anti-rabbit antibody was purchased from EarthOx, LLC (San Francisco, USA). DyLight 448 Affinipure Rabbit Anti-Goat IgG and DyLight 594 Affinipure Goat Anti-Rabbit IgG were purchased from Jacson. Mouse anti-GAPDH antibody and HRP-labeled goat anti-mouse antibody were purchased from Beyotime Institute of Biotechnology (Shanghai, China). ECL Western Blotting Substrate System was purchased from Pierce (Rockford, IL, USA).

### Patients and specimens

Tissue microarrays used in this study were from Liver cancer institue, Zhongshan Hospital. (*Shanghai Super Biotec company (ZL-LVC1604)*). Eighty HCC patients who underwent curative resection, defined as complete macroscopic removal of the tumor, with clinicopathologic and follow-up data were inrolled in this study. None of the patients received anticancer therapy before sampling. The hepatocellular carcinoma diagnosis was established by history, physical examination, computer tomography, MRI (Magnetic Resonance Imaging), liver biopsy and subsequent histological and cytological analyses. Curative resection was defined as complete resection of tumor nodules and the surgical free margin of more than 5 mm by pathological examination; have no cancerous thrombus found in the portal vein(main trunk or two major branches), hepatic veins, or bile duct; and having no extra hepatic metastasis found.

All patients were followed every 3–4 months afterward. Most patients died of intrahepatic recurrence, distal metastasis, or complicated liver cirrhosis. All patients were monitored prospectively by serum AFP, abdomen ultrasonography (US), and chest X-ray every 1–6 months, according to the postoperative time. For patients with test results suggestive of recurrence, computed tomography (CT) and/or magnetic resonance and/or distal metastasis had occurred. Adiagnosis of recurrence was based on typical imaging appearance in CT and/or MRI scan and an elevated AFP level. Overall survival (OS) was defined as the interval between the dates of surgery and death. Disease-free survival (DFS) was defined as the interval between the dates of surgery and recurrence or the last follow-up if no recurrence was observed. Clinical stages of tumors were determined according to the TNM classification system of International Union Against Cancer (Edition 6). Tumor differentiation is grade according to Edmondson - Steiner classification.

### Ethics approval

All samples were coded anonymously in accordance with local ethical guidelines, as stipulated by the Declaration fo Helsinki with written informed consent and a protocol approved by the Ethics Review Committee of Zhongshan Hospital of Fudan University, and every patient provided written informed consent before enrollment.

### In vivo tumorigeniticity

The HCC tumor xenografts animal models were established as follow: 1 × 10^6 Huh7 pSLIK empty vector, Huh7-shNova1 cells, SMMC7721 pSLIK empty vector and SMMC7721-Nova1 cells were inoculated subcutaneously into the right side backs of the nude mice. For the tet-on system [6], Huh7-shNova1 and SMMC7721-Nova1 groups were further randomly divided into two groups, with (+Dox) or without (−Dox). (+Dox: Dox added into the mouse drinking water at a concentration of 1 mg/ml.) After the injection, the tumor size was estimated according to the formula: volume(mm^3^) = 0.5 a^2^ × b (a, major diameter of tumor; b, minor diameter perpendicular to the majour one). Animals were feed for 4 weeks and in the end of feeding, animals were sacrificed. The tumor were removed for western blot analysis.

### Immunochemical staining

The dissected tumor samples for immunohistochemistry were fixed in phosphate-buffered neutral formalin, embedded in paraffin, and cut into 5-μm-thick sections. Five-micron thick sections and tissue microarrays were fixed in 4 % formaldehyde and embedded in paraffin. Tissue samples were deparaffinized and rehydrated, followed by high-temperature antigen retrieval via microwave in 0.1 M citrate solution (pH 6.0) for 15 min. After blocked with 5 % normal goat serum at room temperature for 30 min, the sections were incubated with rabbit anti-human GABA_A_Rγ2 antibody at 4 °C overnight, and then incubated with appropriate secondary antibody at room temperature for 30 min, and finally immunostained by avidin-biotin complex technique using 3,3′-diaminobenzidine. Hematoxylin was used as a counterstain.

The total amount of positive cells in each section was evaluated by two independent investigators masked to clinical outcome and clinicopathologic data. Positive staining cells were observed by using one light microscope (Olympus BX51, Japan). The Friedrichs scoring system was applied to analyze expression of in tumor and peritumor tissues. The intensity of GABA_A_Rγ2 staining was divided into four grades, score “0” for negative, “1” for light yellow, “2” for deep yellow, “3” for brown. GABA_A_Rγ2 staining positive cells was further subgrouped into score “0” for less than 5 %, “1” for 6–20 %, “2” for 21–50 % and “3” for more than 51 %. The total score was defined as staining intensity and scales. Total score higher than 2 is considered as high expression, while under 2 as low expression.

### Western blotting

Total cell or tissue lysates were generated and equal amount protein was subjected to 10 % SDS-PAGE gel, and then transferred onto polyvinylidene difluoride (PVDF) membranes (Millipore, Bedford, USA). Membranes were blocked in blocking solution (50 mM Tri-HCl, 150 mM NaCl, 5 % (w/v) non-fat dry milk and 0.1 % Tween-20) at room temperature for 1 h, followed by incubation with Rabbit anti-human GABA_A_Rγ2 antibody and rabbit anti-human GABA antibody at 4 °C overnight. After three times washing by 0.1 % TBS-Tween20, the membrane was incubated with secondary antibody at room temperature for 1.5 h, the blot were demonstrated by enzyme-linked chemiluminescence using a Fluor Chem FC2 chemilumilescent, fluorescent and visible light gel imaging system (Alpha Inotech, USA).

### Co-immunoprecipitation (co-IP)

The cell-lysated proteins were precleared with 2 μg appropriate antibodies at 4 °C for 8 h, and then incubated with protein G-agarose (Roche) and antibodies at 4 °C overnight. For co-IP experiments using anti-Ub antibody, the cell lysates contained 1 % SDS and received prior heat treatment. The precipitates were pelleted, washed three times with the lysis buffer and analyzed by western blotting.

### Statistical analysis

All values were expressed as mean ± standard deviation (SD). All statistical analyses were performed using the SPSS 18.0 (SPSS Inc., Chicago, IL). *χ* ^2^-test or Fisher’s exact tests was used for the association of GABA_A_Rγ2 expression with the clinicopathologic features. Student’s *t* test and independent sample *t* test were used for comparison between groups. Cumulative survival time was calculated by kaplan-Meier method and analyzed by the log-rank test. Univariate and multivariate analyses were based on the Cox proportional hazard regression model. *P* < 0.05 considered as statistically significant.

## Results

### Nova1 promotes tumor growth in vivo

**Fig. 1:**
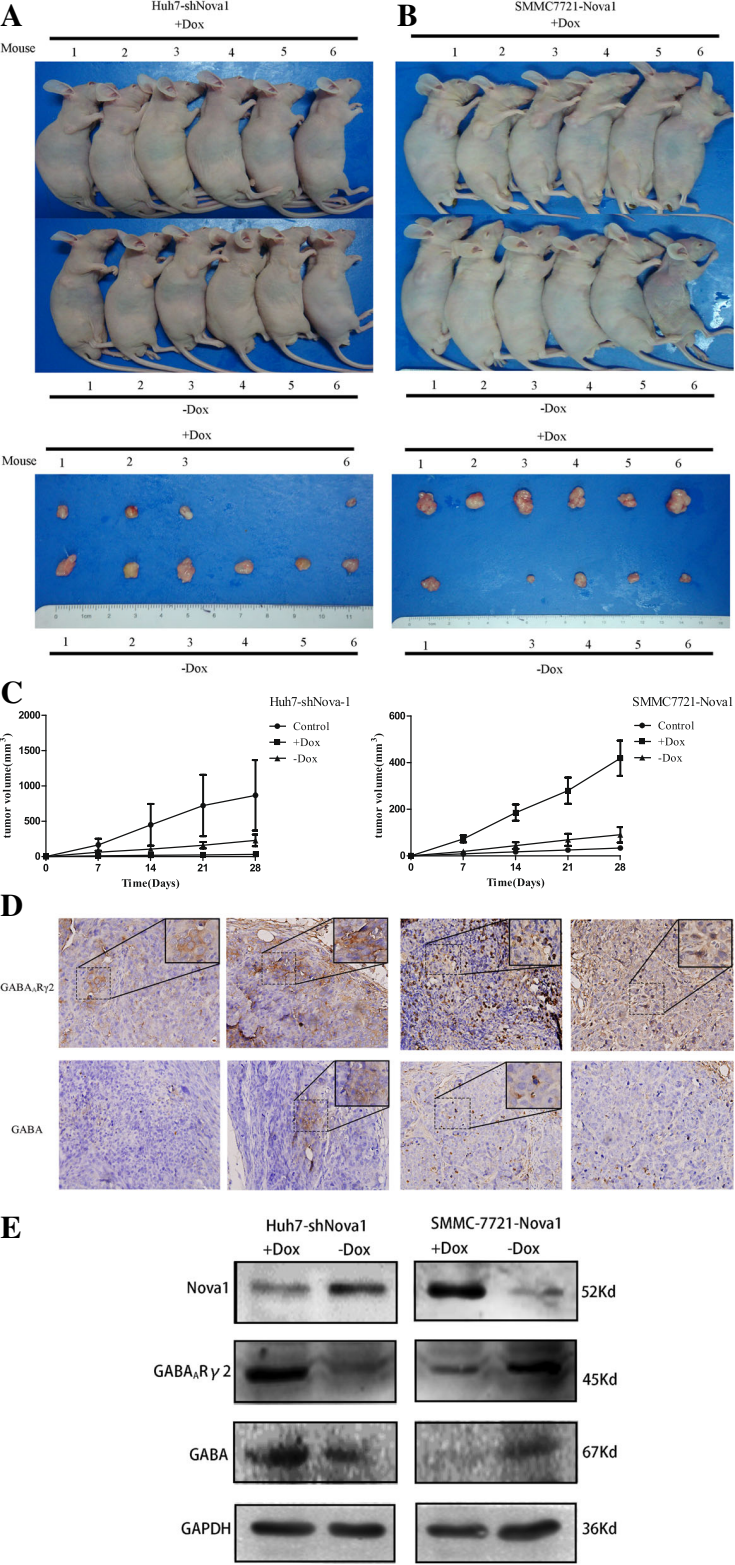
Nova1 promotes tumor growth in vivo and the expression of GABA_A_Rγ2 in tumor tissue is restro-regulated by Nova1. Photography of in vivo tumorigeniticity of Huh7-shNova1 (**a**) and SMMC7721-Nova1 (**b**). **c** Growth kinetics of tumor volumn in nude mice. Tumor diameters were measured every 7 days. **d** Representative photographs of immunohistochemical analysis of GABA_A_Rγ2 and GABA antigens in tumors of nude mice (original magnification:×200; upper right coner,×400). **e** Western blot of the expression of GABA_A_Rγ2 and GABA in tumors of nude mice under the regulation by overexpression or down-regulation the expression of Nova1

As shown in Fig. 1, for Huh7-shNova1, tumor volumes in + Dox group were smaller than that in –Dox group (31.25 ± 15.90 mm3 vs. 287.10 ± 51.10 mm3, *P* < 0.05). While for SMMC7721, the results showed that tumor of + Dox group grew faster and larger than –Dox (418.9 ± 185.6 mm3 vs. 107.7 ± 78.83 mm3, *P* < 0.01). These data indicated that Dox-induced Nova1 expression promotes subcutaneous HCC growth significantly (Fig. 1a-c).

### Expression of GABA_A_Rγ2 and GABA in hepatocellular carcinoma under the regulation of ectopic Nova-1

Later, immunohistochemical staining and western blot were carried out on tumor biopsy of nude mice. Study showed that down-regulation of Nova-1 accompanied with increased expression of GABA_A_Rγ2 and GABA (Fig. 1d-e). The ectopic expression of Nova-1 restro-regulates the expression of GABA_A_Rγ2 and GABA.

### GABA_A_Rγ2 interacts with Nova-1 in peripheral tumor tissues

**Fig. 2:**
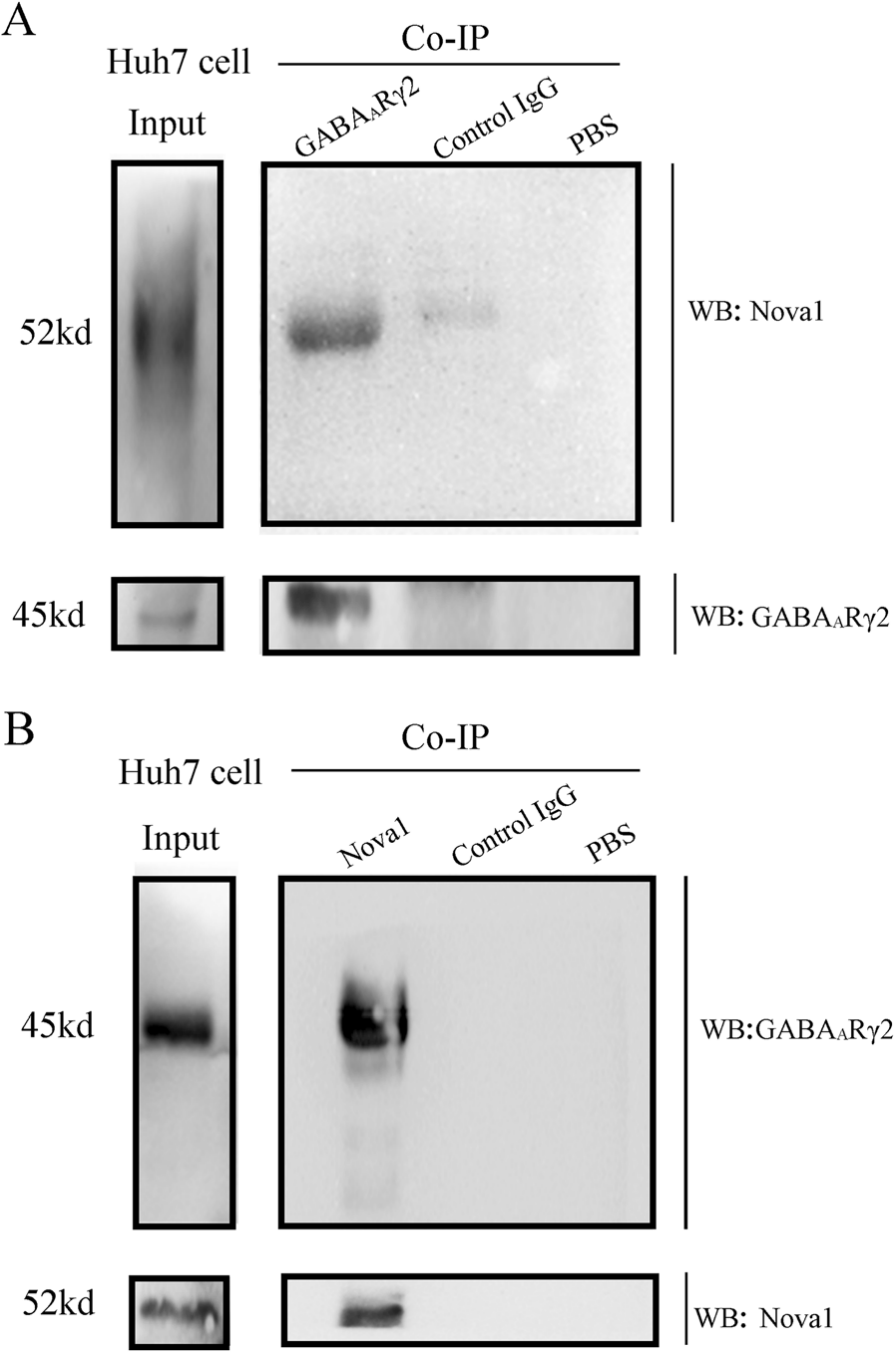
Nova1 interacts with GABA_A_Rγ2 in HCC. For co-IP experiment, total lysates were prepared from Huh7 cells. Co-IP was performed with anti-Nova1 antibody (**a**) and GABA_A_Rγ2 (**b**) antibody. Total lysates were subjected to western blotting with the indicated antibodies as inputs. Parallel expreiments were done in which co-IP antibody was replaced with isotype control antibody igG or PBS

Co-IP was used to verify the possibility of GABA_A_Rγ2 interacts with Nova1 outside the CNS in protein level. In Huh-7 cells, GABA_A_Rγ2 was easy to be detected through anti-Nova-1 antibody. Reciprocal co-IP experiments using anti-GABA_A_Rγ2 antibody indicated the existence of Nova-1. To verify the stringency of co-IP, parallel expreiments were done in which co-IP antibody was replaced with isotype control antibody IgG or PBS. Meanwhile, control experiments showed negative results (Fig. 2).

### Expression level of GABA_A_Rγ2 in HCC patients and its clinical significance

**Fig. 3:**
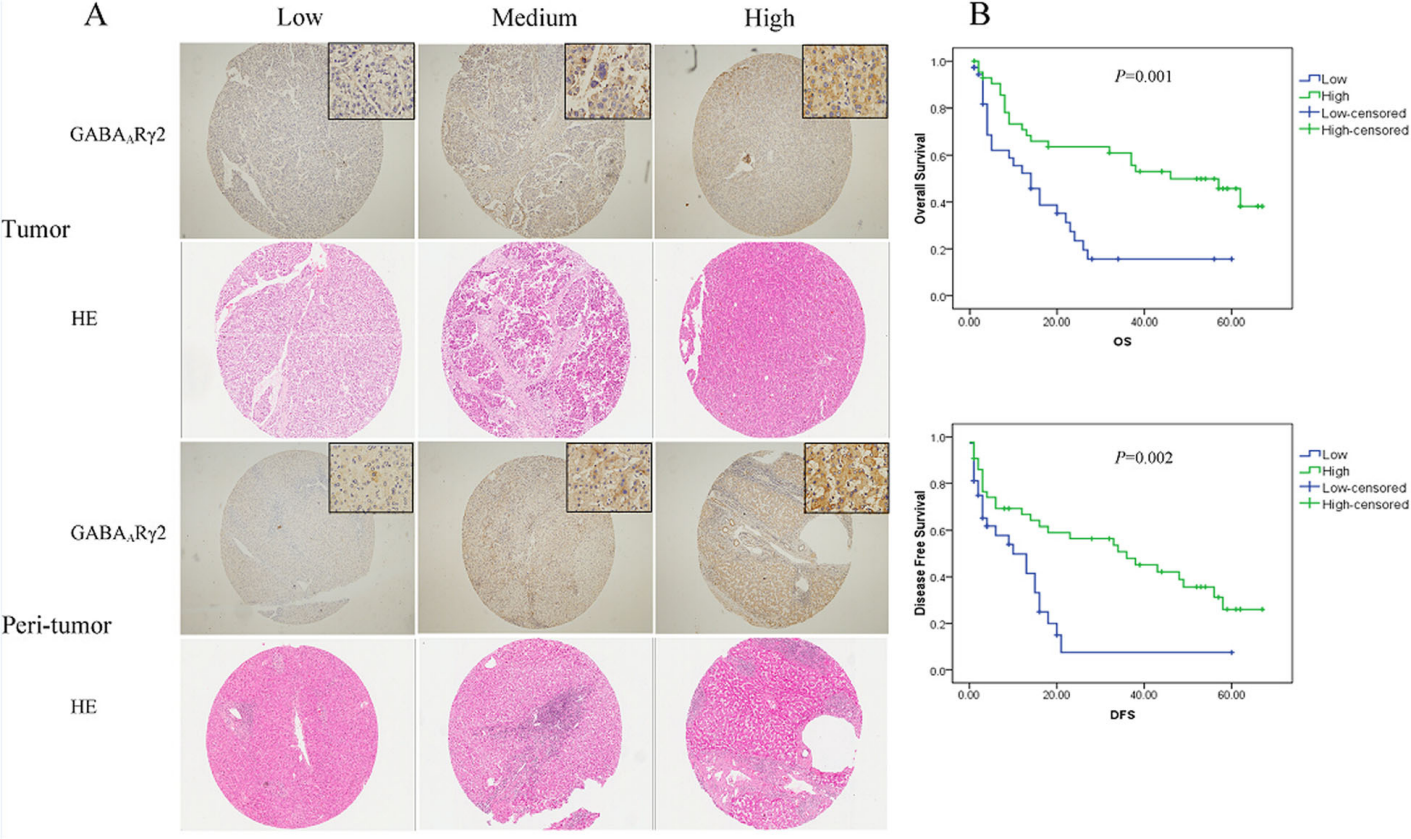
Immunohistochemical analysis of GABA_A_Rγ2 expression in tumor and peritumor tissues on HCC tissue microarrays. **a** Representative immunohistochemical and hematoxylinandeosin (HE) staining images of GABA_A_Rγ2 in tumor and peritumor tissue grouped by negative, moderate, and intense stainings. Original magnification:×40; upper right coner:×400. **b** High expression of tumor GABA_A_Rγ2 correlates with favorable survival rate. Overall survival rate(*Upper*) and disease free survival rate (*Down*) between Patients with high and low expression of GABA_A_Rγ2 were estimated by the Kaplan-Meier method and compared by the log rank test

To explore the occurrence of GABA_A_Rγ2 expression in HCC, we first detected GABA_A_Rγ2 protein levels in tumor and peritumoral tissues. GABA_A_Rγ2 shows a mainly cytoplasmic staining on various normal liver cells and cancer cells (Fig. 3a). The percentage of GABA_A_Rγ2 positivity in the cancerous tissues was lower than that of para-cancerous tissues (35 %, 28/80 vs. 57.5 %,46/80 respectively, *P* = 0.004). As shown in Table 1, clinicopathologic variables such as vascular invasion (*P* = 0.014), tumor number (*P* = 0.016) and TNM (*P* = 0.008) stage were found to be associated with expression levels of intratumoral GABA_A_Rγ2 expression. None of the clinicopathologic variables were associated with peritumoral GABA_A_Rγ2 expression.

**Table 1:** Intratumoral and Peritumoral GABA_A_Rγ2 expression according to characteristics of HCC patients

Characteristics		Intratumoral GABAARγ2			peritumoral GABAARγ2		
		low	high	*p* values	low	high	*p* values
		*n* = 52	*n* = 28		*n* = 34	*n* = 46	
Gender	Male	44	24	0.896	29	39	0.949
	Female	8	4		5	7	
Age(years)	≤53	33	14	0.243	20	17	0.991
	>53	19	14		14	29	
Preoperative AFP(ng/ml)	≤20	15	14	0.06	11	18	0.533
	>20	37	14		23	28	
HBsAg	Negtive	11	10	0.158	7	14	0.322
	Positive	41	18		27	32	
Cirrhosis	No	2	4	0.176^a^	2	4	1.000^a^
	Yes	50	24		32	42	
Vascular invasion	No	12	14	0.014	7	19	0.051
	Yes	40	14		27	27	
Number	Single	36	26	0.016	25	37	0.465
	Multiple	16	2		9	9	
Size(cm)	<5	16	13	0.165	12	16	0.962
	≥5	36	15		22	30	
Tumor Differentiation	I-II	7	4	1.000^a^	4	7	0.751
	III-IV	45	24		30	39	
TNM stage	I	11	14	0.008	7	18	0.077
	II-III	41	14		27	28	
BCLC stage	A	5	6	0.18	3	8	0.338
	B/C	47	22		31	38	

^a^Fisher’s exact test:*χ*2 for all other analyses

On univariate analysis of our data, several clinical factors including liver cirrhosis, vascular invasion, TNM stage and BCLC stage, showed prognostic significance for both OS and DFS (Table 2). Notably, intratumoral GABA_A_Rγ2 expression was a favorable predictor for OS and DFS (HR = 0.372 and 0.419, respectively, *P* < 0.01). Clinicopathologic features showing significance by univariate analysis were adopted for further multivariate cox proportional hazards analyses (Table 3). Liver cirrhosis, Barcelona clinic liver cancer (BCLC) stage and intratumoral GABA_A_Rγ2 expression remained associated with OS (*P* = , 0.01 and <0.001 respectively) while Liver cirrhosis,TNM stage and intratumoral GABA_A_Rγ2 expression remained associated with DFS (*P* = 0.007, 0.035 and 0.002, respectively).

**Table 2:** Univariate analyses of factors associated with survival and recurrence

Variables	OS		DFS	
	Hazard ratio (95 % CI)	*P*	Hazard ratio (95 % CI)	*P*
Sex(male vs female)	1.126 (0.525–2.416)	0.76	1.483 (0.844–2.608)	0.171
Age,y(≥53 vs. < 53)	1.609 (0.902–2.874)	0.107	0.802 (0.361–1.781)	0.588
Preoperative AFP, ng/ml (>20 vs. ≤ 20)	1.320 (0.721–2.415)	0.369	1.390 (0.778–2.485)	0.266
HBsAg (negative vs.positive)	1.175 (0.598–2.311)	0.639	1.040 (0.563–1.925)	0.899
Liver cirrhosis (yes vs. no)	3.303 (1.290–8.455)	0.013	2.878 (1.121–7.385)	0.028
Vascular invasion (yes vs. no)	1.976 (1.004–3.891)	0.049	0.922 (0.523–1.625)	0.78
Tumor size, cm (>5 vs. ≤ 5)	1.107 (0.609–2.014)	0.738	0.818 (0.468–1.429)	0.48
Tumor number (multiple vs.single)	1.718 (0.886–3.330)	0.109	1.265 (0.646–2.475)	0.493
Tumor differentiation (I-II vs.III-IV)	1.215 (0.566–2.608)	0.618	0.971 (0.472–1.999)	0.937
TNM stage (III vs.I-II)	2.253 (1.209–4.199)	0.011	2.137 (1.177–3.880)	0.013
BCLC stage (C vs. A/B)	1.976 (1.004–3.891)	0.049	0.922 (0.523–1.625)	0.78
Intratumoral GABA_A_Rγ2 (high vs.low)	0.372 (0.203–0.683)	0.001	0.419 (0.232–0.755)	0.004
Peritumoral GABA_A_Rγ2 (high vs.low)	1.302 (0.719–2.355)	0.384	1.503 (0.850–2.657)	0.161

**Table 3:** Multivariate analyses of factors associated with OS and DFS

	Hazard ratio (95 % CI)	*P*
OS		
Liver cirrhosis (yes vs. no)	3.711 (1.448–9.510)	0.006
Vascular invasion (yes vs.no)		NS
TNM stage (II-III vs. I)		NS
BCLC stage (B/C vs. A)	2.591 (1.251–5.365)	0.01
Intratumoral GABAARγ2 (high vs.low)	0.309 (0.163–0.588)	<0.001
DFS		
Liver cirrhosis (no vs.yes)	3.334 (1.386–8.020)	0.007
TNM stage (II-III vs. I)	2.009 (1.050–3.844)	0.035
Intratumoral GABAARγ2 (high vs.low)	0.351 (0.183–0.647)	0.002

Since statistic results showed intratumoral GABA_A_Rγ2 was favorable predictor for recurrence, we then analyze this biomarker for early recurrence (metastasis after surgery ≤24 months) together with other clinical feathers that showed prognostic significance for TTR (Table 4). Liver cirrhosis and intratumoral GABA_A_Rγ2 expression were found to be an independent predictor for early recurrence (*P* = 0.046 and 0.01).

**Table 4:** Prognostic factors for early recurrence

Factor	Early recurrence		
	Univariate	Multivariate	
	*P*	HR (95 % CI)	*P*
Liver cirrhosis (yes vs. no)	0.039	2.610 (1.016–6.706)	0.046
Vascular invasion (yes vs.no)	0.197		NS
TNM stage (II-III vs. I)	0.011		NS
BCLC stage (B/C vs. A)	0.197		NS
Intratumoral GABA_A_Rγ2 (high vs.low)	0.022	0.41 (0.208–0.811)	0.01
Peritumoral GABA_A_Rγ2 (High vs. Low)	0.087		NS

Kaplan-Meier analysis and the log-rank test were performed to evaluate the effect of GABA_A_Rγ2 on survaival (Fig. 3b). Patients with higher intratumoral GABA_A_Rγ2 expression had significantly longer OS (median 46, months) or DFS (median, 36, months) than patients with lower intratumoral GABA_A_Rγ2 expression. (median, OS: 14 months;DFS: 10 months, *P* = 0.001 and 0.002 respectively). Peritumoral GABA_A_Rγ2 was not associated with OS or DFS (*P* > 0.05).

## Discussion and conclusions

The GABA_A_ receptor is an ionotropic receptor whose endogenous ligand is GABA, an inhibitory neurotransmitter in the central nervous system [12, 13]. By activation, the GABA_A_ receptor selectively conducts chloride ion influx through its pore, leading to hyperpolarization of the neuron, thus causing an inhibitory effect on neurotransmission [14, 15]. In humans, subunits of GABA_A_ receptor include six types α subunits(α1-α6), three βs(β1-β3), three γs(γ1-γ3), a δ, a ε, a θ and a π [16]. GABA_A_ receptors are most prevalent not only in central nervous system but also in peripheral tissues [8]. By studying the relationship between decreased hepatocyte membrane potential and proliferation of hepatocellular carcinoma, the expression of some receptors in the peripheral tumor such as GABA_A_ receptor-β3 [9], GABA_A_ receptor-α3 [10], and GABA_B_ Receptor family [11] were found to correlate with tumor growth. GABA_A_Rγ2 belongs to the GABA receptor family, however, among all these studies, the potential effects of GABA_A_Rγ2 on tumor proliferation has not been mentioned yet.

In the present study, we found that GABA_A_Rγ2 is expressed mainly in cytoplasm and little in nuclear of HCC by immunohistochemical staining. These findings corresponded with previous several reports proved the expression of GABA_A_ receptors in peripheral tumor tissues, such as receptor α2, β1, β3 and γ3 in human gastric cancer cell KATO III [17], receptor ε and π in human liver cancer tissue [9]. The expression of GABA_A_Rγ2 in tumor tissue is lower than that in paired peritumoral tissues which also be consistent with the research by Gerald Y. Minuk et al. [9]*.* By quantitative reverse transcription polymerase chain reaction (qRT-PCR), they found that GABA_A_-β3 mRNA expression was decreased in tumor compared with adjacent nontumor tissue [9]. These two findings support previous descriptions of down-regulated or absent expression of GABA_A_ receptor in human malignant hepatocyte cell lines and GABA_A_ receptor β3 subunit expression inversely correlated with regenerative activity of human and rat liver [18, 19]. According to our previous study [6], we suppose that the potential mechanism of Nova1 as an oncogene for HCC may due to its interaction with GABA_A_ receptor γ2.

Before that, Dredge et al. found the splicing regulation of the GABA_A_Rγ2 subunit pre-mRNA exon E9 is disrupted in mice lacking Nova1 [7]. Transfection of pNova1-plasmid caused a dose-dependent increase in E9 inclusion in 293 T cells [7]. In CNS, the interaction between Nova1 and GABA_A_ Rγ2 is delineated in the way that Nova1 acts directly on GABA_A_ Rγ2 pre-mRNA by a direct action on clustered YCAY (Y is a pyrimidine) intonic repeats [7]. In our study, Co-IP experiments proved the protein level interaction between GABA_A_Rγ2 and Nova1 in HCC. Furthermore, western blot showed down-regulation of Nova1 accompanied with increased expression of GABA_A_Rγ2 and GABA. The ectopic expression of Nova1 restro-regulates the expression of GABA_A_Rγ2 and GABA. It is unclear why upregulation of Nova1 did not turn out to be an increase expression of GABA_A_Rγ2 in tumor tissue, but restro-regulates the expression of GABA_A_Rγ2 instead. Some mechanisms could partly explain the reason. (1) Dredge et al. found that Nova1 binds with high affinity to the wild-type but not the mutated sequence. Maybe the mutation of these YCAY repeats to YAAY in HCC completely abolished Nova1’s effect on splicing thus leading to the decrease expression of GABA_A_Rγ2 [20, 21]. (2) Gerald Y. Minuk et al. [9] found that the methylation status of GABA_A_ receptor β3 promoter in HCC resulted in attenuated downstream GABA_A_ receptor β3 gene expression. Therefore, the change of the methylation status of the upstream GABA_A_Rγ2 promoter in HCC may also exist. (3) Another view point proposed by Buckanovich et al. is that the interference with the RNA-binding activity of Nova1 may be an immune-mediated rather than the genetic mechanisms. Auto-antibodies due to ectopic expression of Nova1 protein abrogate the Nova-1-RNA interaction via immune-mediated mechanism leading to down-regulaiton of GABA_A_Rγ2 [20]. Since no futher evidence to prove the existence of muation sequence, nor further analysis of the GABA_A_Rγ2 promoter methylation in HCC, nor experiments had been carried out to test the abrogation of auto-antibody of Nova1, additional studies to elucidate the mechanisms involved is needed.

Survival analyses showed that univariate and multivariate analysis disclosed the relationship of intratumoral GABA_A_Rγ2 and OS or DFS in HCC patients that intratumoral GABA_A_Rγ2 was an independent prognostic factor for OS and DFS. Moreover, intratumoral GABA_A_Rγ2 strongly correlated to HCC early recurrence. Some recognized factors such as BCLC stage, TNM stage vascular invasion were not associated with early recurrence, mostly likely because of the limited number of patients enrolled in the study. More HCC patients and longer follow-up study are still requied to verify our results in future study.

In conclusion, we demonstrated an decreased expression of GABA_A_Rγ2 in HCC compaired to adjacent normal tissue. Intratumoral GABA_A_Rγ2 was an independent prognostic factor for OS and DFS. Nova1 interacts with GABA_A_Rγ2 not only in CNS but also in HCC. Over-expression of Nova1 restro-regulates the expression of GABA_A_Rγ2 in HCC. Nova1 promotes tumor growth in vivo and its potential mechanism as an oncogene may due to its interaction with GABA_A_ Receptor γ2. A better understanding of the mechanism of RBP Nova1 for HCC progression might facilitate the design of more effective immunotherapies or gene therapy for hepatocellular carcinoma.

## Abbreviations

AFP: Alpha fetoprotein
BCLC: Barcelona clinic liver cancer
CI: Confidence interval
CNS: Central nervous system
DFS: Disease free survival
GABA_A_: γ-aminobutyric acid_A_
GABA_A_Rγ2: GABA_A_ Receptor-γ2
HCC: Hepatocellular carcinoma
NS: Not significant
OS: Overall survival
PBS: Phosphate-buffered saline
qRT-PCR: Quantitative reverse transcription polymerase chain reaction
RBP: RNA-binding proteins
TNM: Tumor-node-metastasis
TTR: Time to recurrence
